# Hyperplasia of lymphoid structures in the hypopharynx: a case report

**DOI:** 10.1186/1752-1947-4-388

**Published:** 2010-11-30

**Authors:** Yuh Baba, Yasumasa Kato, Kaoru Ogawa

**Affiliations:** 1 Department of Otorhinolaryngology, Ohtawara Red Cross Hospital, 2-7-3 Sumiyoshi-cho Ohtawara City, Tochigi 324-8686, Japan; 2 Department of Biochemistry & Molecular Biology, Kanagawa Dental College, Yokosuka 238-8580, Kanagawa, Japan; 3 Department of Otorhinolaryngology, Head and Neck Surgery, Keio University, 35 Shinanomachi Shinjuku, Tokyo 160-0082, Japan

## Abstract

**Introduction:**

Ectopic tonsillar tissue simulating a benign tumor of the hypopharynx is described in this report.

**Case presentation:**

We report the case of a 79-year-old Japanese woman with globus sensation. Because clinical observation revealed benign features, tumor tissue was laryngoscopically resected. From the pathological features, for example, existence of germinal center, lymphoid tissue, and crypt involving lymphoepithelial symbiosis, we diagnosed that the lesion was a hyperplasia of ectopic tonsillar tissue.

**Conclusions:**

Although ectopic tonsillar tissue of the hypopharynx is relatively rare, it should be kept in mind during differential diagnosis.

## Introduction

As many kinds of benign tumors, malignant tumors, and tumor-like lesions may occur in the hypopharynx (YB *et al*. unpublished data) it may be difficult to establish a clinical diagnosis. Here, we report a hypertrophied lymphoid case of the hypopharynx which was histologically diagnosed as the hyperplasia of ectopic tonsillar tissue. The tissue was removed surgically under general anesthesia. After the surgery, there was no obvious symptom of recurrence during, at least, the following year.

## Case presentation

**Figure 1:**
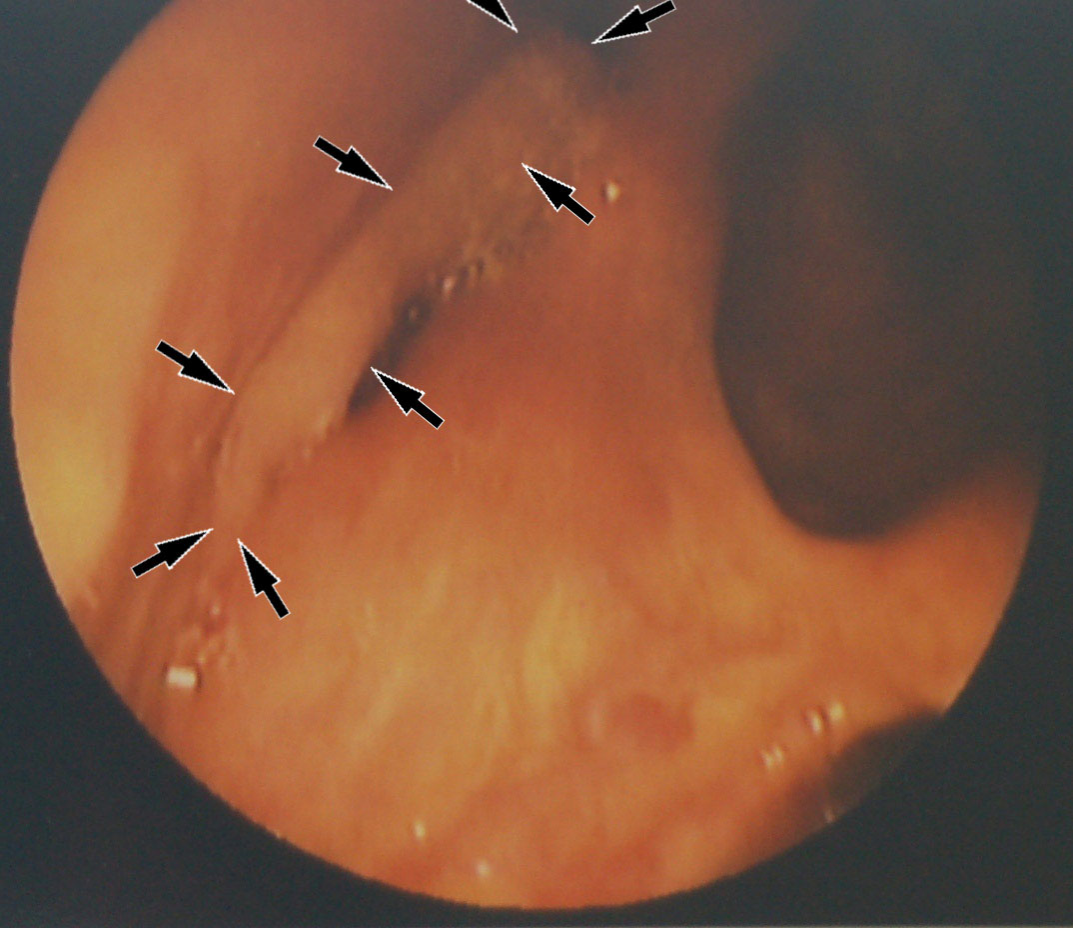
**Location of the ectopic tonsillar tissue in the right pyriform recess of the hypopharynx**.

An otherwise healthy 79-year-old Japanese woman visited our department on 22^nd ^July 2008. She had a medical history of chronic pharyngolaryngitis. The patient first visited her general practitioner because she had been aware of a foreign body sensation in her throat since December 2007. However, her condition did not improve, although she was treated by her general practitioner. She, therefore, visited our hospital in July 2008 to ask for a second opinion. When the first medical examination was performed by us using laryngopharyngeal fiberscopy, we observed a smooth mucosal swelling in the right pyriform recess but no vocal cord fixation (Figure 1). Computed tomography (CT) revealed no space-occupying lesion in the right pyriform recess (data not shown). From these observations, a preoperative diagnosis of a benign tumor was made. The swelling was restricted to the surface of the hypopharyngeal mucosa.

**Figure 2:**
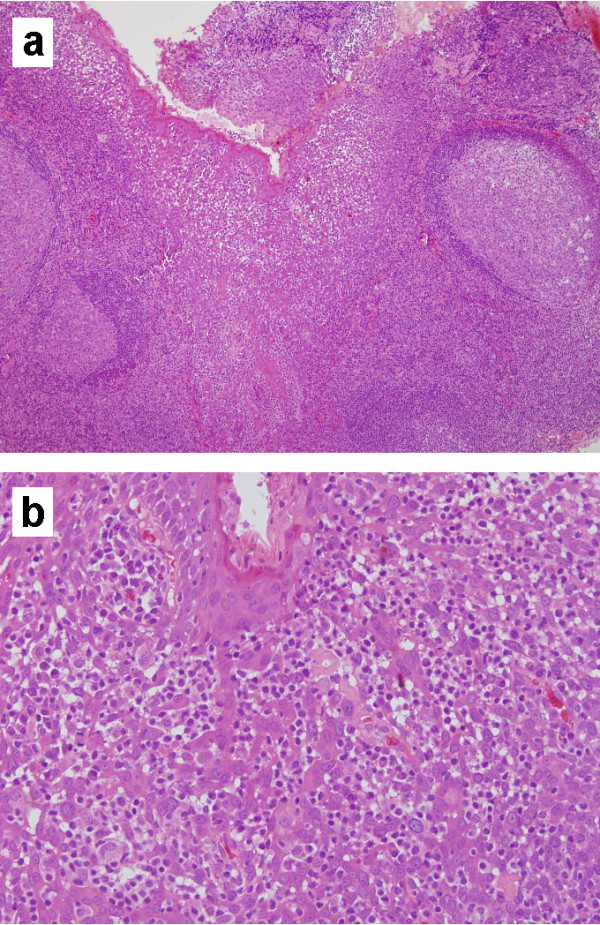
**Pathological features**. **(a) **Germinal center, lymphoid tissue, and crypt were noted. HE, X10 **(b) **Ectopic tonsillar tissue showing lymphoepithelial symbiosis in crypt. HE, X40

On 18 September 2008, the tissue was resected laryngoscopically under general anaesthesia and this sample was pathologically analyzed. We observed histological features of hyperplastic tonsil, for example, existence of germinal center, lymphoid tissue, and crypt involving lymphoepithelial symbiosis (Figure 2). An ectopic tonsil is tonsillar tissue that was developed in areas outside the palatine, tongue, pharynx, and Eustachian tube. Therefore, we diagnosed that the lesion was a hyperplasia of ectopic tonsillar tissue in the hypopharynx. The postoperative course was uneventful. Follow-up examinations showed there was no sign of recurrence of the swelling or complications during the following year.

## Discussion

Although benign tumors such as papilloma or adenoma can generally occur in the hypopharynx, we found tonsillar tissue in this lesion. Ectopic tonsil is tonsillar tissue that was established at areas outside of the four major tonsil groups, which include the palatine tonsil, the lingual tonsil, the pharyngeal tonsil, and the tubal tonsil. The tonsil is a lymphoid tissue with a germinal center and crypt involving lymphoepithelial symbiosis. In our case we observed these criteria.

The pathogenesis of our case is not clear. However, it is reported that ectopic tonsillar tissue in the nasal septum may be the result of a persistent infection [1]. Our patient had a medical history of chronic pharyngolaryngitis. Taken together, the patient^'^s history of chronic laryngophatyngitis may have induced hyperplasia of the lymphoid tissues, in addition to the ectopic aberration. Furthermore, we observed that a tonsillar cyst is located in the pyriform recess [2]. It was proposed that an oral cyst originated from an ectopic oral tonsil and obstruction of the crypt caused a cyst to form because of the persistent infection [3]. This proposal is consistent with our speculation with regard to the pathogenesis of this case. This resected tissue may be the precursor of the tonsillar cyst in the pyriform recess.

A few reports on ectopic tonsillar tissue have been published, showing that ectopic tonsillar tissues have been reported to be in the nasal septum [1], the floor of the mouth [3-6], the orbit [7], the ventral surface of the tongue [3,4,6], the soft palate [3,4], and the larynx [8]. A search of the literature suggests that ectopic tonsillar tissue of the hypopharynx is relatively rare. These lesions can be asymptomatic and easily overlooked, making them appear even rarer.

## Conclusions

Although ectopic tonsillar tissue of the hypopharynx is relatively rare, it should be included in the differential diagnosis of patients who are aware of a foreign body sensation in their throats.

## Abbreviations

CT: computed tomography

## Consent

Written informed consent was obtained from the patient for publication of this case report and any accompanying images. A copy of the written consent is available for review by the Editor-in-Chief of this journal.

## Competing interests

The authors declare that they have no competing interests.

## Authors^, ^contributions

YB assisted in the conception and design of the paper, and also helped in the acquisition, review and interpretation of the data. YK contributed towards data collection and drafting of the manuscript. KO was involved in conception of the paper, and in reviewing and finally approving the version of the manuscript to be published. All authors read and approved the final manuscript.
